# Acute Pulmonary Embolism in COVID-19: A Report of Two Cases

**DOI:** 10.7759/cureus.9459

**Published:** 2020-07-29

**Authors:** Yashvin Onkarappa Mangala, Saravgunjit Singh Daid, Seetha Lakshmanan, Rahul Kapil, John Miskovsky

**Affiliations:** 1 Internal Medicine, Roger Williams Medical Center, Providence, USA; 2 Internal Medicine, Roger Williams Medical Center, Boston University School of Medicine, Providence, USA

**Keywords:** acute pulmonary embolism, covid 19, anticoagulation, venous thromboembolism (vte)

## Abstract

Coronavirus disease 2019 (COVID-19), which is currently causing a global pandemic, is found to be associated with abnormal coagulation parameters and hyper-coagulable state with increased risk of venous thromboembolism (VTE). Here, we present two non-ICU cases of COVID-19, complicated with acute pulmonary embolism (PE). As of now, there are no proper guidelines established on anticoagulation in these patients. We discuss the pathophysiology and management strategy based on recently published studies on anticoagulation in COVID-19 patients.

## Introduction

Coronavirus disease 2019 (COVID-19), caused by the novel coronavirus (SARS-CoV2), is a highly contagious disease that was initially identified in Wuhan, Hubei province of China in December 2019 [1]. It has caused a global pandemic affecting 188 countries and jeopardizing healthcare systems around the world [2]. 

Although knowledge of this novel coronavirus is emerging, the most common cause for hospitalization of COVID-19 patients is severe respiratory distress. Among those hospitalized, the morbidity and mortality are increased due to a number of complications including coagulation abnormalities [3-4]. We present two cases of COVID-19 pneumonia complicated by acute pulmonary embolism (PE). 

## Case presentation

Case 1

**Figure 1:**
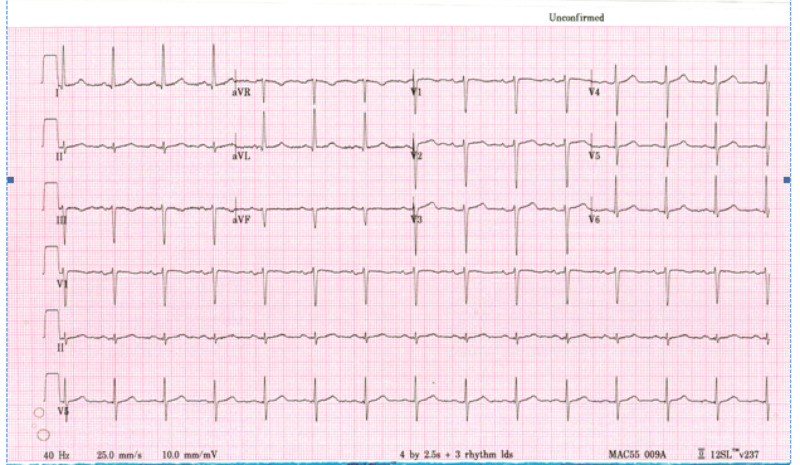
EKG of Case 1 showing normal sinus rhythm. EKG, electrocardiogram

**Figure 2:**
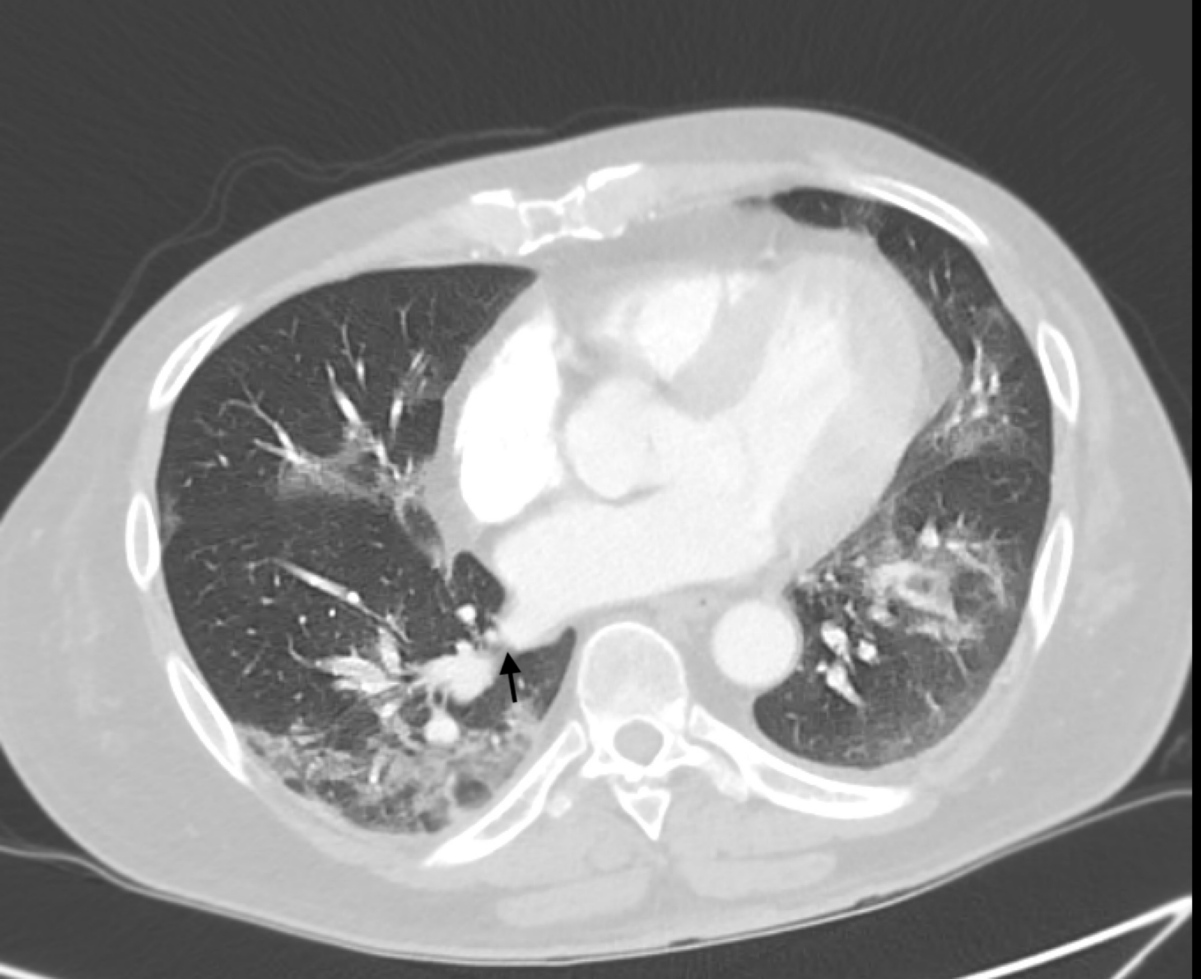
CT angiography of the chest revealing pulmonary emboli in the right lower lobe.

A 55-year-old male who was initially admitted to the alcohol detoxification unit at our hospital reported having a nonproductive cough and diarrhea on day 2 of admission. He was found to have a fever of 101.5℉. However, he denied any chest pain or difficulty breathing. He was transferred to the medical floor and started on empiric antibiotics for suspected aspiration pneumonia with piperacillin-tazobactam and azithromycin. Nasopharyngeal sampling was done to test for COVID-19 and found to be positive. Inflammatory markers were markedly elevated with C-reactive protein (CRP) on admission at 31.31 mg/dL peaking to 78.57 mg/dL, d-dimer peaked at 4.05 mg/L, lactate dehydrogenase (LDH) peaked at 361 IU/L, and ferritin of >1499 ng/mL. Antibiotics were then stopped and he was started on hydroxychloroquine and prophylactic anticoagulation with enoxaparin 40 mg once daily. On day 4 of hospitalization, the patient reported a sudden onset of shortness of breath associated with central chest pain. Initial workup with an electrocardiogram (EKG) and troponin was negative but CT imaging of the chest with contrast revealed multiple PE in the right lower lobe (Figures 1-2). Therapeutic anticoagulation was initiated with enoxaparin 90 mg twice daily. The patient required intermittent nasal cannula oxygenation during his stay, however, his clinical condition gradually improved along with down-trending of inflammatory markers. Enoxaparin was transitioned to apixaban, and the patient was discharged home with appropriate follow up.

Case 2

**Figure 3:**
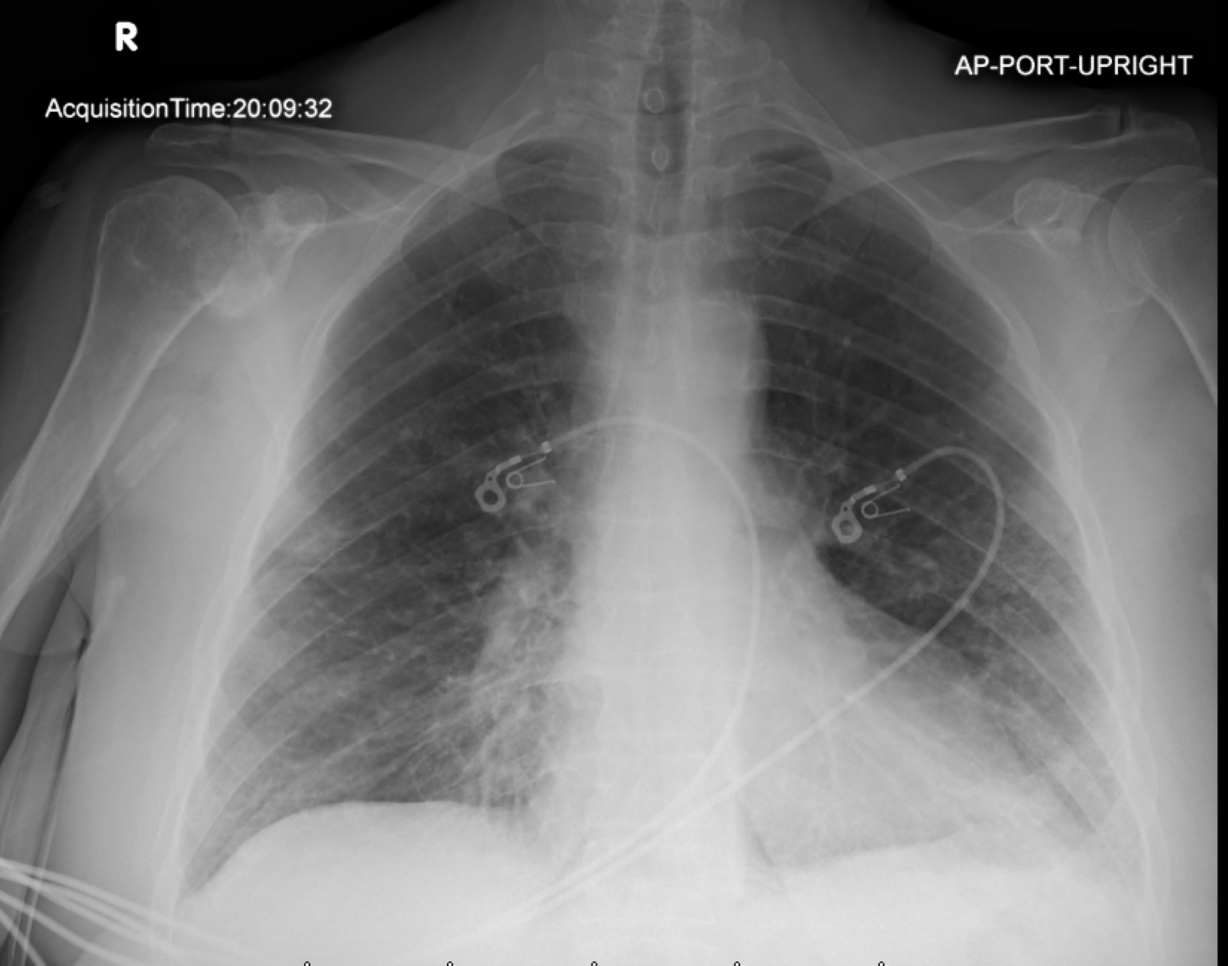
Initial Chest X-ray showing right mid-lung and bi-basilar airspace disease.

**Figure 4:**
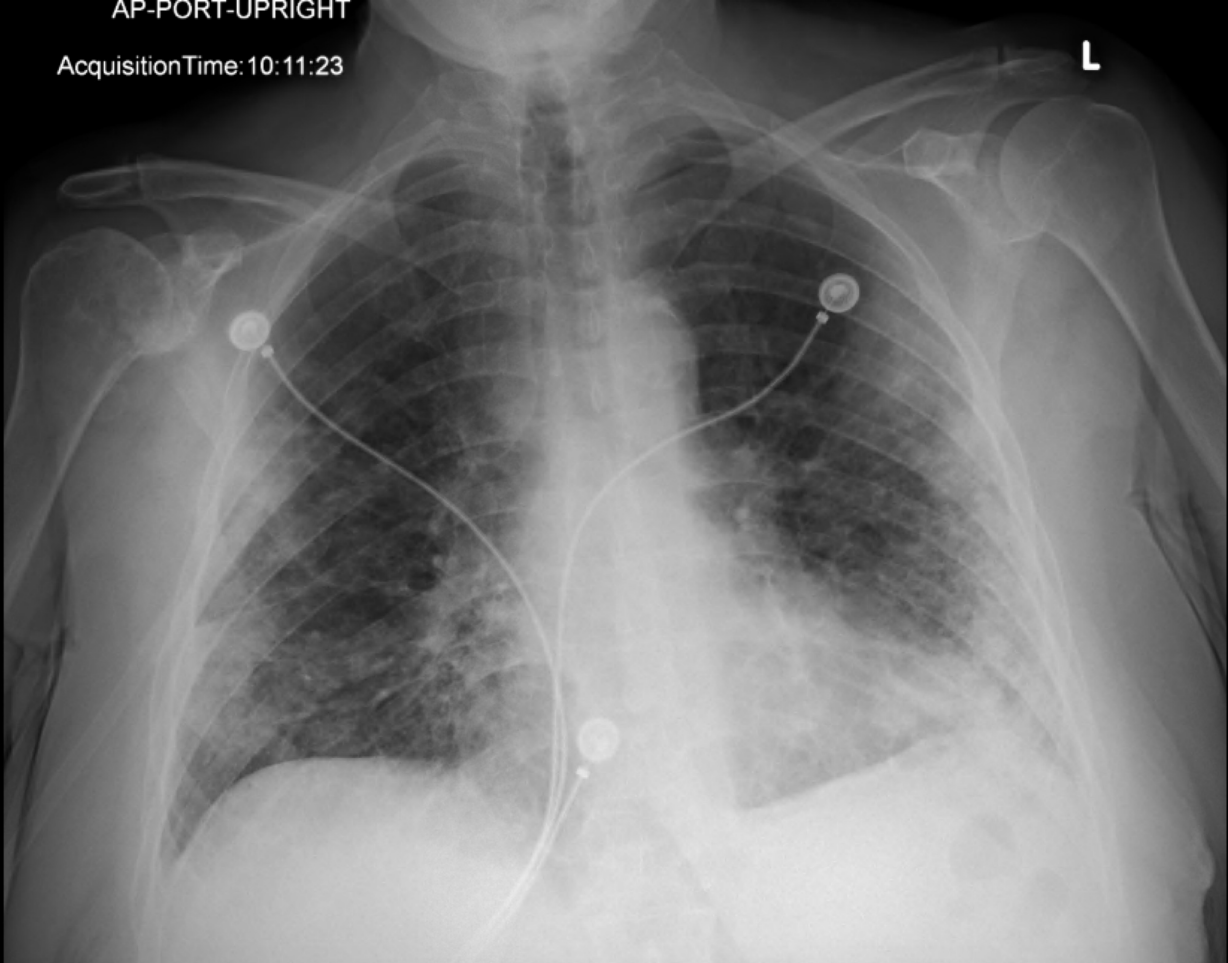
Repeat chest X-ray showing persistent multifocal airspace disease with interval worsening in aeration.

**Figure 5:**
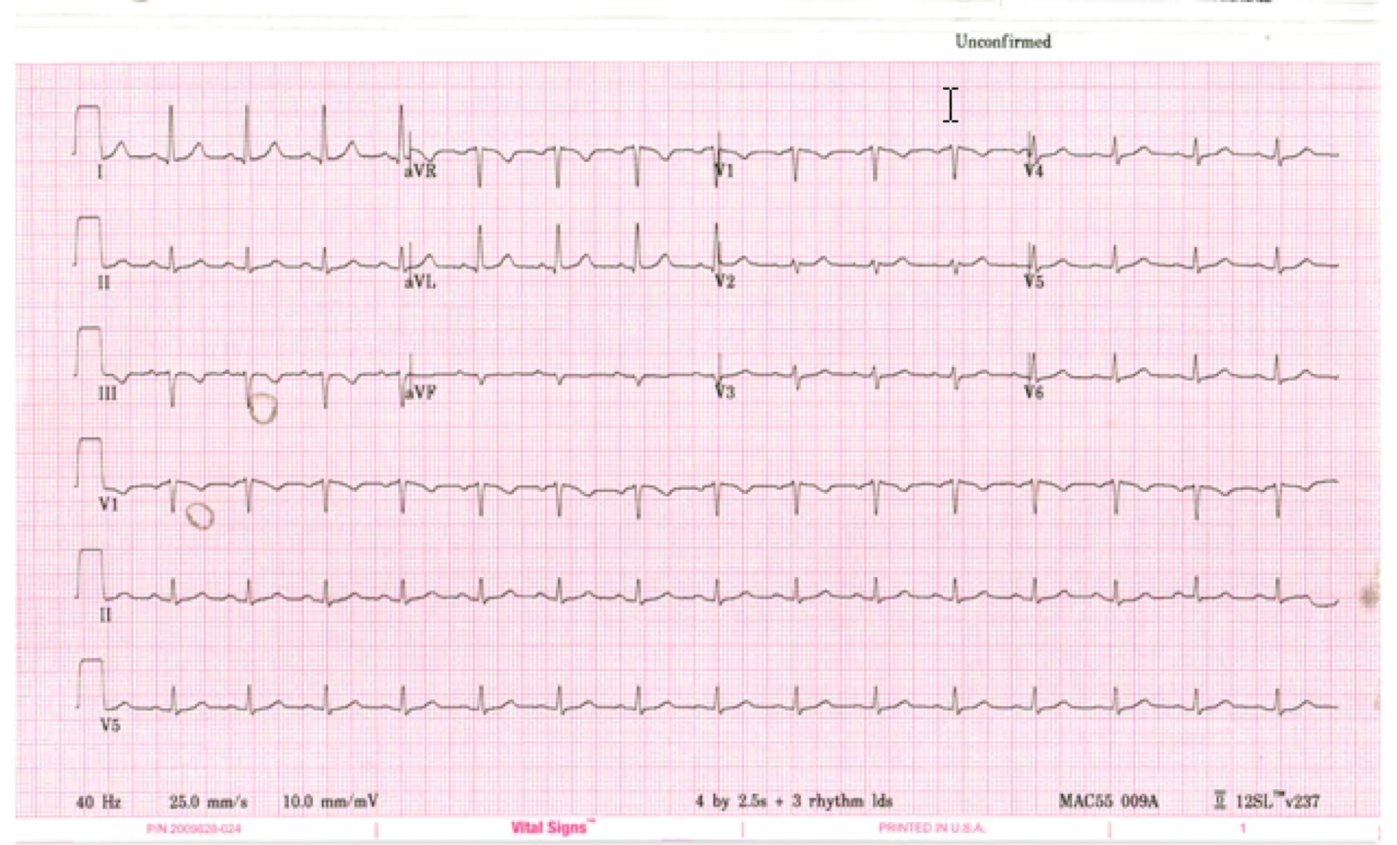
EKG of Case 2 showing normal sinus rhythm. EKG, electrocardiogram

**Figure 6:**
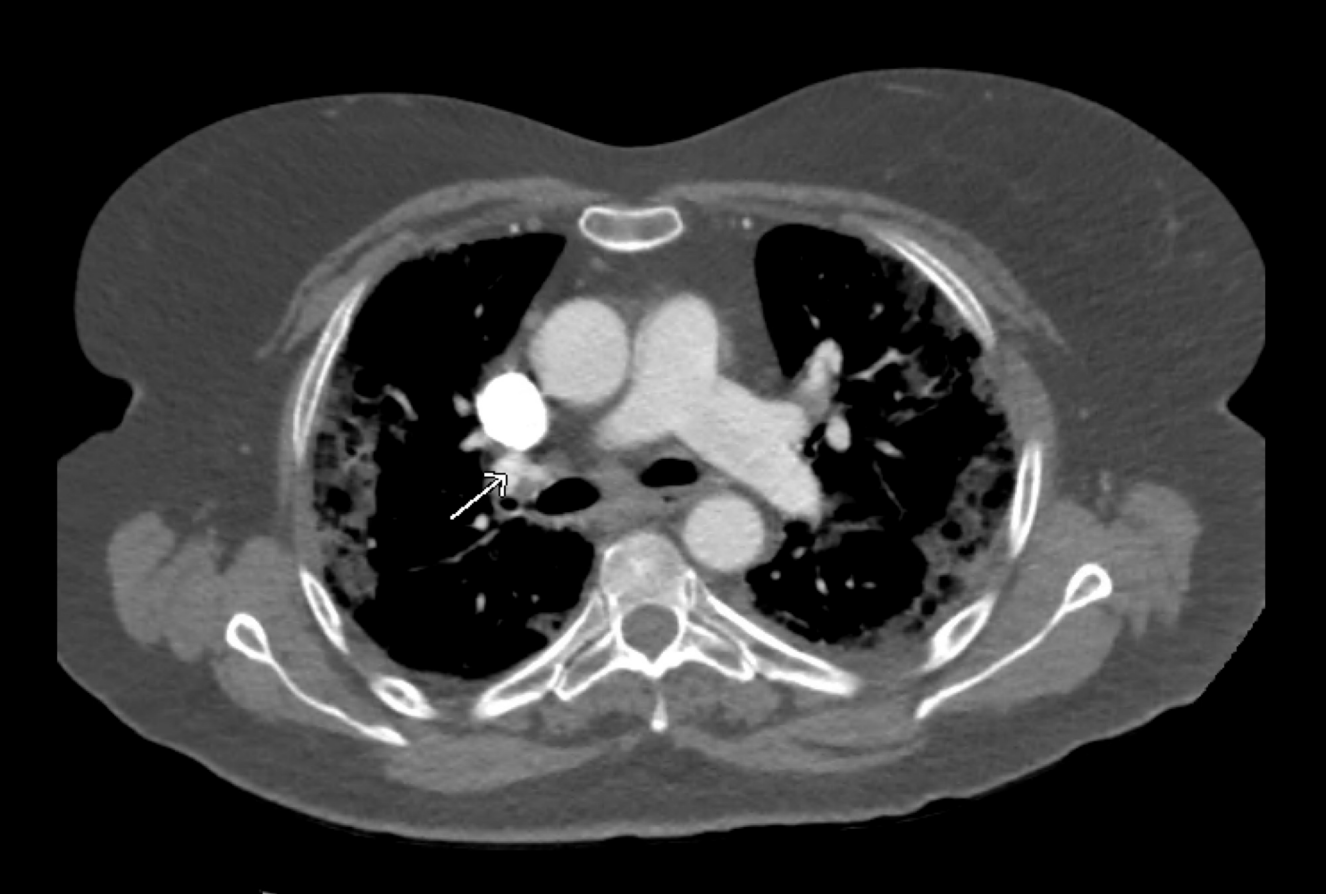
CT angiography of chest showing pulmonary emboli in right upper lobe pulmonary artery and segmental branches of right lower lobe pulmonary artery.

A 67-year-old female with a medical history of hypertension and stable rheumatoid arthritis on methotrexate, leflunomide, and hydroxychloroquine presented to our facility with worsening dry cough, fever, and dyspnea for five days. She was tested for COVID-19 by her primary care physician after a telemedicine evaluation the week prior, which resulted positive. In the ED, she was found to be febrile with a temperature of 102℉. Initial laboratory workup revealed a white blood cell (WBC) count of 4100/mm3 with mild lymphopenia, erythrocyte sedimentation rate (ESR) of 25 mm/h, CRP of 31.63 mg/L, and ferritin of 827.7 ng/mL. A chest X-ray showed right mid-lung and bi-basilar airspace disease (Figure 3). The patient’s methotrexate and leflunomide were stopped, however, she was continued on hydroxychloroquine. She was also given azithromycin and zinc for COVID-19 pneumonia and enoxaparin 40 mg daily for prophylactic anticoagulation. The patient continued to have high-grade fevers and reported worsening shortness of breath over the next few days. Repeat chest radiograph showed a slight worsening of airspace disease, while her EKG and troponin were unremarkable (Figures 4-5). D-dimer was elevated at 1.34 ng/mL and subsequent chest CT angiography showed findings of PE involving right upper lobe pulmonary artery and segmental branches of right lower lobe pulmonary artery (Figure 6). She was started on therapeutic anticoagulation with IV heparin for the treatment of acute PE. Over the next few days, the patient’s clinical condition stabilized with down-trending of inflammatory markers. She was eventually discharged home on apixaban with appropriate follow up.

## Discussion

Majority of patients with severe COVID- 19, evidenced by acute lung injury or acute respiratory distress syndrome, have elevated d-dimer and fibrinogen levels that are supportive of hyper-coagulable state from cytokine storm syndrome [5]. These coagulation abnormalities are associated with poor outcome in severe COVID 19 patients [6]. The true incidence of venous thromboembolism (VTE) in COVID-19 patients is currently unknown as the COVID-19 pandemic continues to evolve. Patients with severe COVID-19 have a higher risk of VTE than those with mild or asymptomatic disease, due to prolonged hospitalization with ICU stay along with traditional risk factors such as male sex, old age, obesity, cancer, and history of VTE. Among the two cases of PE described in this article, there was no family/personal history of VTEs or recent surgery/immobilization. 

Endothelial dysfunction caused by infection may result in excessive thrombin generation and fibrinolysis shutdown [7]. Endothelial cell activation or damage due to virus attachment to the ACE2 receptor with microvascular thrombosis is thought to play an important role in the pathogenesis of hypoxemic respiratory failure [8-9]. Also, hypoxia in severe COVID-19 can stimulate thrombosis through the hypoxia-inducible transcription factor-dependent signaling pathway [10]. This is concordant with the recent autopsy studies that have reported occlusion and micro-thrombus formation in small pulmonary vessels of critical patients with COVID-19 [11]. There have been reports of concurrent PE during the SARS outbreak in 2004 and the H1N1 influenza pandemic in 2009 [12-13]. Murine models suggest that SARS-CoV may interact with urokinase to produce a hyper-coagulable state as previously observed in the SARS-related acute lung injury [14]. 

A recently published Dutch study of 184 COVID-19 patients in the ICU, reported 25 confirmed cases of PE, one deep vein thrombosis, and two catheter-related thromboses [15]. These patients were on nadroparin thromboprophylaxis, ranging from 2850 IU once daily to 5700 IU once daily (enoxaparin 40 mg is equivalent to nadroparin 4000 IU). There are, however, no reported studies on the VTE rate in outpatients or non-ICU inpatients. 

The guidelines on optimal thromboprophylaxis in COVID-19 are still emerging. International Society on Thrombosis and Hemostasis (ISTH) advocates the use of laboratory tests, including d-dimers, prothrombin time, and platelet count to stratify patients at risk of adverse outcomes and who need hospital admission [16]. Drug-drug interactions between antiviral treatments and direct oral anticoagulants (DOACs) and difficulty in maintaining stable INRs in vitamin K antagonists during acute illness pose a huge challenge in their utilization. Patients who were previously on DOACs or vitamin K antagonists should be switched to either low molecular weight heparin (LMWH) or unfractionated heparin (UFH) upon hospitalization with COVID-19 [17]. COVID-19 patients with presumed or documented PE need to continue therapeutic anticoagulation for a minimum duration of three months, followed by discontinuation if they have no ongoing risk factors for thrombosis or other indications such as atrial fibrillation [18].

## Conclusions

In summary, it is recommended that patients requiring hospital admission for COVID-19 pneumonia be started on prophylactic anticoagulation with LMWH or un-fractionated heparin to prevent thromboembolism, in the absence of contraindication. In the case of elevated d-dimer levels on admission or sudden clinical worsening, CT pulmonary angiography should be considered as PE is a life-threatening but potentially treatable condition.
